# Light-Sheet Fluorescence Microscopy with Scanning Non-diffracting Beams

**DOI:** 10.1038/s41598-020-63847-2

**Published:** 2020-05-22

**Authors:** Hosein Kafian, Meelad Lalenejad, Sahar Moradi-Mehr, Shiva Akbari Birgani, Daryoush Abdollahpour

**Affiliations:** 1 Department of Physics, Institute for Advanced Studies in Basic Sciences (IASBS), Zanjan, 45137-66731 Iran; 2 Department of Biology, Institute for Advanced Studies in Basic Sciences (IASBS), Zanjan, 45137-66731 Iran; 3 Optics Research Center, Institute for Advanced Studies in Basic Sciences (IASBS), Zanjan, 45137-66731 Iran

**Keywords:** Imaging and sensing, Microscopy, Light-sheet microscopy

## Abstract

Light-sheet fluorescence microscopy (LSFM) has now become a unique tool in different fields ranging from three-dimensional (3D) tissue imaging to real-time functional imaging of neuronal activities. Nevertheless, obtaining high-quality artifact-free images from large, dense and inhomogeneous samples is the main challenge of the method that still needs to be adequately addressed. Here, we demonstrate significant enhancement of LSFM image qualities by using scanning non-diffracting illuminating beams, both through experimental and numerical investigations. The effect of static and scanning illumination with several beams are analyzed and compared, and it is shown that scanning 2D Airy light-sheet is minimally affected by the inhomogeneities in the samples, and provides higher contrasts and uniform resolution over a wide field-of-view, due to its reduced spatial coherence, self-healing feature and longer penetration depth. Further, the capabilities of the illumination scheme is utilized for both single-and double-wavelength 3D imaging of large and dense mammospheres of cancer tumor cells as complex inhomogeneous biological samples.

## Introduction

Light-sheet fluorescence microscopy (LSFM) as a novel noninvasive method provides the possibility of *in*-*vivo* volumetric imaging from the depth of thick samples, while retaining high spatial and temporal resolution simultaneously, with a minimized photodamage and phototoxicity^1^. LSFM is rapidly becoming a unique technique for functional neuroimaging in large networks^2–4^, and presents the possibility of morphological imaging of a whole cleared mammalian brain, with sub-cellular resolution^5^. In contrary to the conventional wide-field and confocal fluorescence microscopes, in LSFM instead of illuminating a tiny region around the focal plane, a wide layer of the sample is illuminated, and emitted fluorescence from the whole illuminated plane is captured all at once. Moreover, utilization of two separate objectives for illumination and detection results in decoupled axial and transverse resolutions, and therefore, enables the capability of three-dimensional (3D) imaging of various large and tiny specimens such as animal embryos^6^, a whole mouse brain^5^, and neuronal activity in a single neuron^7^. Moreover, the method is used for developmental studies in biology^8^ and also for clinical histopathology of large specimens^9^. However, it should be noted that the spreading of the light-sheet and its deterioration, due to scattering and absorption, severely limit the capability of imaging large and dense specimens. For instance, to achieve a micron-scale axial resolution, a conventional Gaussian illuminating beam has to be focused to a micron-thick light-sheet, leading to a profound action of diffraction and faster spreading of the light-sheet around the confocal region. Thus, the optimum axial resolution will be limited only to a few microns in the transverse plane around the focus within the confocal range, leading to a blurry image around the focus. Furthermore, in the process of illuminating a sample with weakly absorbing or inhomogeneous structures, the light-sheet is severally disturbed by the emergence of a stripe pattern caused by the interference of various parts of the excitation beam after passing through inhomogeneities or absorbing structures in the sample. This effect leads to non-uniform illumination of the sample and results in artificial structures, also known as “ghost image”, in the captured images^10^. As a matter of fact, this effect is a crucial challenge in LSFM and therefore several approaches have been suggested for diminishing the issue. Rotating the sample for capturing images with different illumination directions^6^, simultaneous illumination from opposite directions^11,12^, and using virtual light-sheets, formed by scanning the illuminating laser beam in a plane perpendicular to the detection direction, were initially proposed for this purpose. The first two approaches although lead to generation of uniform light-sheet in a wider field-of-view (FOV) and mitigates the ghost image, inevitably result in longer exposure of the sample to exciting illumination and therefore may induce photodamage, and also have rather complex experimental arrangements. Using non-diffracting beams such as Bessel beam^13–15^, modified Bessel beam^16^, and Airy beams^17–19^ have also been proposed for this purpose. Although, the self-healing property of the non-diffracting beams is expected to enhance the uniformity of the illumination light-sheet, their mutli-lobe intensity structure leads to simultaneous illumination of various depths, and reduces the image contrast. Therefore, finding an optimum illumination scheme for achieving high quality images with minimized artifacts, and also an enhanced resolution and contrast over a wide FOV, in large and thick samples is a severe issue to be addressed concretely. Recently, Fahrbach *et al*. have numerically studied axial resolution in LSFM using various forms of illumination light-sheets in^20^.

Here, we experimentally and numerically investigate the role of illumination on the quality of the images captured by light-sheet fluorescence microscopy. We analyze and compare the LSFM images with several static and scanning light-sheets formed by different beams such as Gaussian, Bessel and Airy beams. Our results clearly reveal that illumination by the scanning 2D Airy beam results in optimal image qualities with minimized effect of the ghost image and high contrast. Additionally, the capability of the illumination scheme is demonstrated through volumetric imaging of large and dense tumorspheres of breast cancer cells.

## Theoretical Background and Numerical Investigations

**Figure 1:**
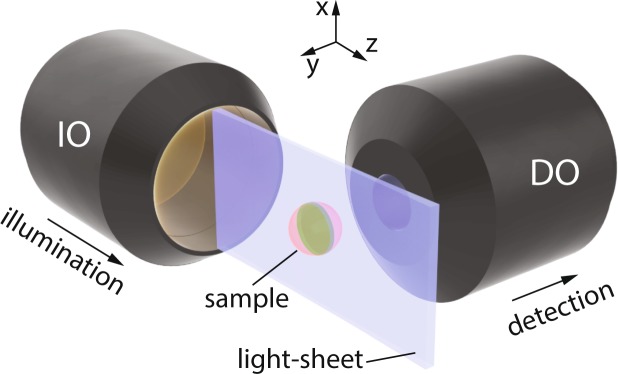
Schematics of the working principle of light-sheet fluorescence microscopy. Sample, located at the vicinity of the focal plane of the illumination objective (IO), is illuminated by a static or a scanning light-sheet, leading to a fluorescence emission from the excited layer whose emission is collected by the detection objective (DO), orthogonally-oriented with respect to the direction of illumination.

The working principal of light-sheet microscopy is shown in Fig. 1. A light-sheet formed by an illumination objective (IO) in the $x$-$z$ plane, illuminates a thin layer of a specimen placed around the focal plane of the objective. The excited layer emits fluorescence that is collected by a detection objective (DO) (perpendicularly oriented with respect to the light-sheet), and an image is captured from the whole illuminated layer at once. Then, the light-sheet or the sample are displaced with respect to each other to capture images from different depths of the sample to create 3D images. Theoretically, if the fluorophore distribution in the sample is denoted by $f(x,y,z)$, and the intensity distribution of a very thin illumination light-sheet (at a depth of $y={y}_{0}$) is represented by ${I}_{{\rm{ill}}}(x,y={y}_{0},z)$, the emitted fluorescence intensity distribution can be described as ${I}_{{\rm{f}}}=f(x,y,z)\cdot {I}_{{\rm{ill}}}$, and incoherent image formation is expressed as

1 $${I}_{{\rm{image}}}(x,y,z)={I}_{{\rm{f}}}(x,y={y}_{0},z)\,\ast \,{{\rm{PSF}}}_{{\rm{\det }}}(x,y={y}_{0},z),$$

where ${{\rm{PSF}}}_{{\rm{\det }}}$ denotes the point spread function (PSF) of the detection microscope, and the symbol $\ast$ indicates two-dimensional convolution operation. Thus, for the very ideal case of using a thin light-sheet (that is also uniform over the FOV of the detection microscope), and when the DO and IO are fixed at their position and only the sample is displaced for capturing images from various depths, the fluorophore distribution in 3D can be accurately retrieved by performing a deconvolution operation on the captured images with a given ${{\rm{PSF}}}_{{\rm{\det }}}$ at the depth of illumination. In such an ideal case the axial resolution of the LSFM is solely determined by the thickness of the light-sheet, while the lateral resolution is only determined by the resolving power of the detection microscope. However, in a more realistic case, for a finite thickness of the light-sheet, that is not necessarily uniform over the FOV, the axial and lateral resolutions will not be uniform over the FOV. For instance when a Gaussian beam is focused by a high numerical aperture (NA) objective, to form a thin light-sheet at the waist of the illumination, the best axial resolution is only achieved within the confocal region of the focused beam and beyond that region the axial resolution is degraded due to the natural spreading of the focused beam. Similarly, the lateral resolution beyond the confocal region of the illuminating beam is also reduced since the thicker illuminated layer may lay out of the depth-of-focus of the detection microscope. Nevertheless, for a finite and uniform thickness of the light-sheet across the FOV, the image formation can be described as^21^

2 $${I}_{{\rm{image}}}(x,y,z)=f(x,y,z)\,\ast \,{{\rm{PSF}}}_{{\rm{sys}}}(x,y,z),$$

where ${{\rm{PSF}}}_{{\rm{sys}}}={I}_{{\rm{ill}}}\cdot {{\rm{PSF}}}_{{\rm{\det }}}$ denotes the PSF of the system that can either be numerically calculated or experimentally measured, for a given illuminating beam and a specific detection system^21^. Finally, in order to retrieve the 3D fluorophore distribution, the captured image has to be deconvolved with the ${{\rm{PSF}}}_{{\rm{sys}}}$, using an appropriate deconvultion algorithm. Therefore, a uniform resolution over the FOV can either be achieved by using a relatively thick conventional Gaussian light-sheet (focused by a low-NA objective), or by using a thin light-sheet formed by non-diffracting beams and their modified forms. Additionally, the emergence of the artifacts caused by inhomogeneities in the samples is expected to be more profound when the illumination is spatially coherent (i.e. in the from of static illuminating light-sheets) since the scattered beam, from the inhomogeneous structures, coherently superpose with the unscattered parts of the beam and lead to the generation of a modulated intensity profile in the form of stripes. On the other hand, when the light-sheet is formed by scanning a beam in a transverse plane ($x$-$z$ plane in Fig. 1) and the camera is set to integrate over several scanning cycles (to form a virtual light-sheet), the area behind the obstacles are illuminated from different directions and hence the uniformity of the illumination is enhanced. Moreover, it is anticipated that the self-healing feature of the non-diffracting beams may suppress the artifacts and provide longer penetration depths of illumination in the samples, eventually leading to higher image qualities in two-and three-dimensions. Nevertheless, non-diffracting beams are composed of an intense main lobe accompanied by low-intensity structures that act like an energy reservoir for the main structure in a way that the power flux from the surrounding structures towards the main lobe during the propagation provides the invariant propagation and also robustness upon encountering obstacles or inhomogeneities in the propagation medium. However, when using these beams for creating light-sheets in LSFM, the fluorescence emissions from several depth, simultaneously excited by the supporting structures of the beams, may also reach the detector and deteriorate the image contrast. Therefore, one must consider a tradeoff between the artifacts caused by the obstacles when using conventional light-sheets, on one hand, and reduction of the image contrast as a result of simultaneous illumination of various depths when using non-diffracting light-sheets, on the other hand. For a through investigation of the effect of the illuminating light-sheet we conducted numerical simulations of the LSFM, based on 3D beam propagation method (BPM)^22,23^ with several static and scanning illumination light-sheets. Several non-fluorescent micro-beads with a diameter of 4.4 *μ*m were embedded in a uniform fluorescent medium. To simulate an inhomogeneous specimen, the refractive indices of the surrounding medium and the micro-beads were set to 1.4 and 1.6, respectively. It should be noted that using non-fluorescent micro-beads embedded in fluorescent surrounding medium has previously been used for numerical and experimental investigation of LSFM image quality factors^10,13^. Different illuminating beam profiles including Gaussian, cosine, Bessel, 1D- and 2D-Airy beams with the same widths of the main lobe (2.4 *μ*m full-width at half-maximum (FWHM), at the beam waist), similar number of side lobes for the cosine, Bessel and Airy beams, and a wavelength of $\lambda =473\,{\rm{nm}}$ were used for generating three static (Gaussian, cosine, and 1D Airy), and three scanning light-sheets (Gaussian, Bessel and 2D Airy). The beam profiles are presented in Supplementary Fig. 1. Static light-sheets were created by a 1D Gaussian beam, a cosine beam (as the 1D counterpart of the Bessel beam), and an exponentially truncated Airy beam, that are localized along *y*–axis, but uniformly extended along *x*–axis. The scanning light-sheets were formed by gradually displacing the center of a Gaussian beam focused by a spherical lens and the core (main lobe) of the Bessel and 2D Airy beams along *x*–axis. Additionally, all of the beams were arranged in such a way that their waist were located in the middle of the FOV along *z*–axis (Supplementary Fig. 2). The micro-beads were placed at different depths in the sample as depicted in Supplementary Fig. 3(a–c), so that the effect of simultaneous illumination of various depths by the side structures of the cosine, Bessel and Airy beams could be assessed as well. The static and scanning light-sheets were propagated through the micro-beads and 3D beam propagation profiles were recorded in the same three-dimensional domain for all beams. Besides, in order to account for the fact that the spheres were not fluorescing, the 3D intensity profiles were set to zero over the volume of each sphere. Eventually, the recorded 3D intensity profiles were convolved with ${{\rm{PSF}}}_{{\rm{\det }}}$ calculated for an imaging system with ${\rm{NA}}=0.42$ at a wavelength of 515 nm based on the Born-Wolf model^24^. Calculated detection PSF is illustrated in Supplementary Fig. 4. Further details of the numerical investigations are provided in the Methods section.

**Figure 2:**
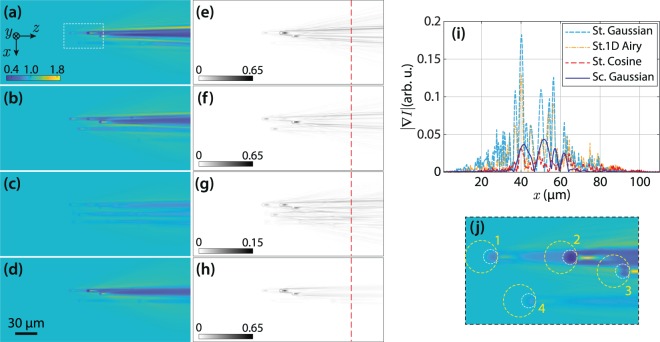
Numerically simulated LSFM images of non-fluorescent micro-beads embedded in a uniform fluorescent medium, with a static Gaussian (**a**), static 1D Airy (**b**), static cosine (**c**), and scanning Gaussian (**d**) light-sheets; images are individually normalized to their uniform fluorescent background. (**e**–**h**) Corresponding intensity gradient (modulus) of (**a**–**d**). (**i**) Line profiles of the intensity gradient over the indicated dashed lines in (**e**–**h**) along $x$-axis. (**j**) A magnified view of the dashed rectangle in (**a**); four particles are labeled, and the areas used for the calculation of local contrast are indicated by yellow dashed circles. The beads are located at several depths, and the beams have identical widths at the middle of the field-of-view.

Simulated LSFM images of the sample for static Gaussian, static cosine, static 1D Airy, and scanning Gaussian light-sheets are shown in Fig. 2(a–d). In order to assist a visual assessment, all images are individually normalized to their background fluorescent intensity. Moreover, for a quantitative assessment of the severeness of the stripes after the particles, the modulus of the 2D intensity gradient, in the form of $|\nabla I|=\sqrt{{\left(\frac{\partial I}{\partial x}\right)}^{2}+{\left(\frac{\partial I}{\partial z}\right)}^{2}}$,was calculated for all images. Higher values of the gradient modulus would indicate stronger appearance of the stripes. The map of the intensity gradient modulus of the images are presented in Fig. 2(e–h), and their line profile, at a $z$ position indicated by the dashed lines in (e–h), are shown in Fig. 2(i). Obviously, the stripes are more profound in the case of the static Gaussian, and 1D Airy light-sheets because of the coherence of the illumination, that leads to further enhancement of the stripes through interference of scattered and unscattered light from the particles. The strength of the stripes are considerably reduced both for static cosine and scanning Gaussian light-sheets; however, the origins of the reduction for the two cases are basically different. In the case of the cosine light-sheet (Fig. 2(c)), the symmetric multi-lobe beam profile along *y*–axis results in simultaneous illumination of various *y*–planes (both before and after the focus plane of the detection), and hence the shadows of the particles in each plane are compensated by the fluorescent light originating from other illuminated planes; this eventually leads to reduced appearance of the stripes. On the other hand, in the case of scanning Gaussian light-sheet, (Fig. 2(d)), as a virtual light-sheet, the interference patterns are mostly averaged out and therefore the stripes are considerably reduced. This simply implies that using a virtual light-sheet is significantly reducing the stripe artifacts.

**Figure 3:**
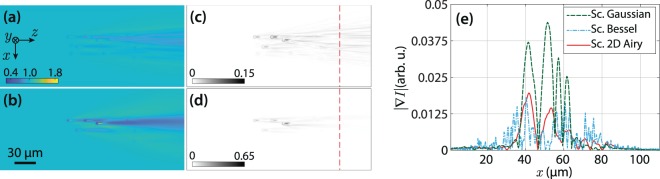
Numerically simulated LSFM images of non-fluorescent micro-beads embedded in a uniform fluorescent medium, with a scanning Bessel (**a**), and a scanning 2D Airy (**b**) light-sheets. (**c** and **d**) Present corresponding intensity gradient (modulus) of (**a**), and (**b**), respectively. (**e**) Line profiles of the intensity gradient over the indicated dashed lines in (**c** and **d**) along $x$-axis. For a comparison a line profile of the intensity gradient modulus corresponding to scanning Gaussian light-sheet is plotted as well. The beads are located at several depths, and the Bessel and 2D Airy beams have identical widths at the middle of the field-of-view, and a comparable number of side lobes.

Moreover, the simulated LSFM images of the scanning Bessel, and 2D Airy light-sheets are presented in Fig. 3(a,b). The modulus of the intensity gradient of the images are shown in Fig. 3(c,d), respectively. Besides, line profiles of the intensity gradient at the same $z$ position are plotted in Fig. 3(e) along with that of the scanning Gaussian light-sheet. Additionally, the standard deviation (SD) of the modulus of the intensity gradient for the six different light-sheets over the line profiles are given in Table 1. The results evidently show that the stripe artifacts are considerably reduced when using the static cosine, scanning Bessel, and 2D Airy light-sheets. However, another main factor in assessing the quality of the captured images in LSFM is the contrast (visibility) of the beads with respect to the background. In order to quantitatively evaluate the contrast of the particles in the images, four beads at different 3D positions were selected. The selected beads are labeled with numbers 1–4 in Fig. 2(j), and also in Supplementary Fig. 3(a,b). An ~80 *μ*m^2^ circular region around each particle, without including the corresponding stripes (as depicted by the dashed circles in Fig. 2(j)), was selected to calculate the local contrast as

3 $${\rm{contrast}}=100\times \frac{{I}_{{\rm{\max }}}-{I}_{{\rm{\min }}}}{{I}_{{\rm{\max }}}+{I}_{{\rm{\min }}}},$$

where ${I}_{{\rm{\max }}}$ and ${I}_{{\rm{\min }}}$ are the maximum and minimum intensities in the selected region, respectively. The values of the local contrast of the selected beads for various illumination light-sheets are given in Table 2. The data explicitly imply that the images with static Gaussian, static 1D Airy, scanning Gaussian and scanning 2D Airy light-sheets provide higher contrasts for all particles. It should be noted that the beads with the static cosine and scanning Bessel light-sheets are observed with the least contrast, although the stripe artifacts are significantly reduced in those cases. The minimal contrast in the case of static cosine and scanning Bessel light-sheets, is due to the fact the simultaneous illumination of various *y*–planes by the symmetric multi-lobe structures of the light-sheets contribute in image formation, and eventually reduce the contrast of the objects. On the other hand, contrast of the beads with scanning 2D Airy light-sheets is comparable with those of the static Gaussian, static 1D Airy, and scanning Gaussian light-sheets, while the deterioration of the image by the stripe artifact is minimal in comparison to those cases.

**Table 1:** Standard deviation of the modulus of the intensity gradient over the line profile for images with different light-sheets.

Illumination light-sheet	SD × 100
Static Gaussian	3.082
Static 1D Airy	2.045
Static cosine	0.642
Scanning Gaussian	1.117
Scanning Bessel	0.363
Scanning 2D Airy	0.440

**Table 2:**
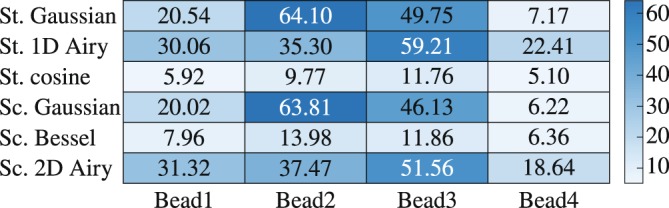
Local contrast of the micro-beads with different illumination light-sheets in the simulated LSFM images.

Additionally, in a more realistic numerical investigation of the LSFM, we also performed simulations in which the refractive indices and sizes of the micro-beads were different. In this case, particle diameters, and refractive indices were randomly selected in the range of 4.4–13.2 *μ*m, and 1.5–1.7, respectively. In Supplementary Fig. 3(d–f), particle size distribution, their positions in three-dimensions, and also their refractive index distributions, are shown in different views. These simulations are performed with scanning Gaussian, Bessel, and 2D Airy light-sheets with the same parameters as the previous simulations. The simulated LSFM images for the three scanning light-sheets are presented in Supplementary Fig. 5(a–c), and their intensity gradient modulus are shown in Supplementary Fig. 5(d–f). Line profiles of the intensity gradient modulus over the dashed lines in (d–f) are plotted in Supplementary Fig. 5(g). Calculated local contrast for three particles (as illustrated in Supplementary Fig. 5(h)), with the three different light-sheets are given in Supplementary Fig. 5(i). In a perfect agreement with the previous simulations, these results also clearly show that the scanning Gaussian light-sheet provides highest contrast while it is maximally affected by the stripes. The image with the scanning Bessel light-sheet is minimally affected by the stripes, but the contrast of the particles are remarkably decreased. Most importantly, in the image with the scanning 2D Airy light-sheet, the artifacts are greatly suppressed, while a high contrast, comparable to the ones with the scanning Gaussian light-sheet, is maintained.

Our numerical simulations vividly indicate that although the static light-sheets (except the cosine light-sheet) provide enhanced visibility in the images, they are significantly affected by the inhomogeneities in the samples. In the case of the cosine light-sheet, the artifacts and also the contrast of the particles are both markedly reduced. Thus, for an experimental investigation of the quality of the LSFM images with different light-sheets we limited the study to scanning light-sheets.

## Experimental Implementation of the LSFM

**Figure 4:**
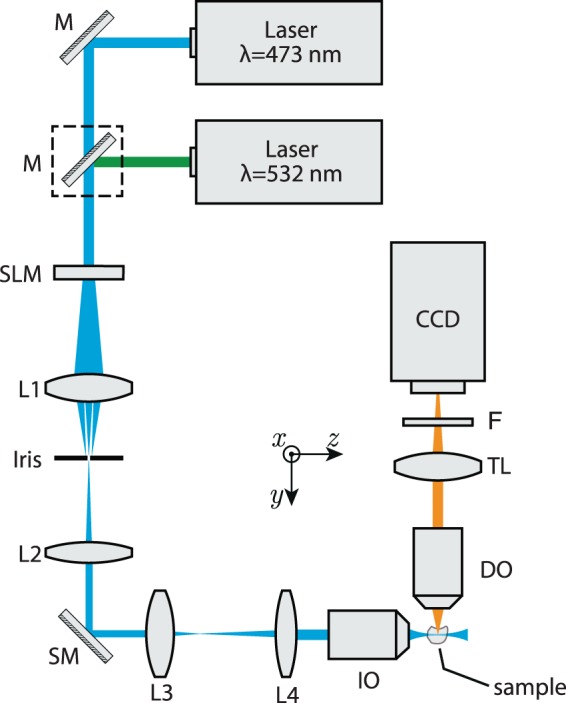
Schematic of the experimentally developed LSFM. The collimated laser beam(s) are incident on a SLM, for imprinting a desired phase profile, and then imaged into the focal plane of the illumination objective (IO) by two 4*f*-systems composed of the lenses L1, L2, L3, and L4. The scan mirror (SM), located at the back focal plane of the IO, provides the beam scanning capability along *x*–axis, for the generation of scanning light-sheets. The waist of the light-sheets overlap with the middle of the field-of-view (FOV) of an orthogonally oriented detection microscope, comprising a detection objective (DO), an infinity-corrected tube lens (TL), a long-pass filter (F) and a CCD camera. For excitation with 532 nm, a mirror (shown in the dashed box) was placed in front of the second laser.

In order to experimentally investigate the role of illumination in light-sheet microscopy, we developed a LSFM system as illustrated in Fig. 4 and used it to image non-fluorescent micro-beads embedded in a fluorescent gel. The output of a continuous wave (CW) diode-pumped solid state laser system (${\lambda }_{1}=473\,{\rm{nm}}$) was collimated to an intensity FWHM of 7.5 mm, and incident on a transmissive liquid-crystal spatial light modulator (LC-SLM) with 1024 × 768 pixels, pixel pitch of 13 *μ*m and maximum phase modulation depth of $\pi$. An additional CW laser system with a wavelength of ${\lambda }_{2}\,=532\,{\rm{nm}}$ with the same FWHM could also be independently used for excitation of fluorescence when needed. The SLM was mainly used for generating various illuminating beam profiles using appropriate phase distributions. The main diffraction order of the SLM was allowed to pass an iris located at the focal plane of a lens L1, and then relayed to the scanning mirror (SM) by a 4*f* system composed of lenses L1 (${f}_{1}\,=30\,{\rm{cm}}$) and L2 (${f}_{2}\,=15\,{\rm{cm}}$). The first 4*f* system also shrinks the beam size by a factor 2 from the plane of the SLM to the plane of the SM. An additional 4*f* system composed of lenses L3 (${f}_{1}\,=10\,{\rm{cm}}$) and L4 (${f}_{2}\,=30\,{\rm{cm}}$) was used to expand and relay the beam to the back focal plane of the illumination objective (IO) (UPLAN, 10×, NA = 0.3, Olympus). The lenses in both relaying systems were chosen in a way to use the full numerical aperture of the IO.

Various phase profiles were prepared and imprinted on the SLM for the generation of several light-sheets with different beam profiles. For the generation of a 2D Airy beam, a phase profile in the form of $\alpha ({x}^{3}+{y}^{3})+\beta (x+y)$ was imprinted on the SLM; where, $x$ and $y$ are the transverse coordinates in the plane of the SLM, and $\alpha$ and $\beta$ are arbitrary cubic and linear phase coefficients, respectively. In the case of the 2D Airy beam, the plane of the SLM was directly relayed onto the back focal plane of the IO by which a 2D spatial Fourier transformation was performed, and the beam was generated at the working distance (WD, ${\rm{WD}}=10\,{\rm{mm}}$) of the IO. On the other hand, for the generation of a Bessel beam, a conical phase profile in the from of $\gamma \rho$ was imprinted on the SLM, where $\rho =\sqrt{{x}^{2}+{y}^{2}}$ and $\gamma$ is an arbitrary coefficient determining the width of the core of the Bessel beam. In this case, the beam was formed immediately after the SLM, and therefore an additional spherical lens, L5 ($f=8\,{\rm{cm}}$), located 6.5 cm before the SM, was used to convert the beam to a ring-shaped beam, through a 2D Fourier transformation, and the resultant beam was then expanded by the second 4*f* system and relayed to the back focal plane of the IO, and eventually transformed back to a Bessel beam at the front focal plane of the IO. Finally, for the generation of a Gaussian beam with a comparable width, the illumination scheme illustrated in Fig. 4 was modified by placing a lens L6 ($f=20\,{\rm{cm}}$) at a distance of 4 cm before L2.

With such an arrangement, and by separately adjusting the parameters $\alpha$ and $\gamma$, a Gaussian beam, a Bessel beam, and a 2D Airy beam with almost identical main lobe intensity FWHMs of ~2.4 *μ*m were formed at the vicinity of the WD of the IO. The generated beam profiles (normalized to their respective maximum intensity) are depicted in Supplementary Fig. 6, where it is seen that the main lobe widths of the beams and also the number of the side lobes of the Bessel and 2D Airy beams are comparable. It should be noted that the modification of the experimental setup, by adding different lenses for generating different illumination beam profiles, leads to a slight displacement of the beam formation distance from the IO (about 1 mm deviation at maximum), that was compensated by moving the object and detection microscope along the propagation direction of the illumination in each case.

Moreover, in order to form the scanning light-sheets, the scanning mirror (SM) was set to oscillate at a proper frequency and amplitude, to generate smooth light-sheets with a dimension of 150 *μ*m along *x*–axis, and 290 *μ*m along *z*–axis. Additionally, for the case of the 2D Airy beam, the side lobes of the beam were extended along the *y*–axis and the generated light-sheet had a parabolic trajectory with a maximum deviation of 3.25 *μ*m over 290 *μ*m in the sample. Moreover, the waist of the light-sheets (along the propagation direction of the illumination), were adjusted to locate at the middle of the FOV of the detection microscope.

The sample was prepared by mixing and steering an aqueous suspension of nonfluorescent silica micro-beads with a diameter of 4.4 *μ*m, in a homogeneous solution of fluorescein and gelatin. The solution was poured in a cubic transparent quartz cuvette with a dimension of ($1\,{\rm{cm}}\times 1\,{\rm{cm}}\times 4\,{\rm{cm}}$), and allowed to gradually solidify in such a way that micro-beads were firmly embedded in the fluorescent gel. The sample was mounted on a three-dimensional motorized translation stage (MT3-Z8, Thorlabs) and the emitted fluorescence from the illuminated layers of the specimen was collected and imaged by a detection microscope orthogonally oriented with respect to the illumination arm. The detection microscope was constituted of a detection objective (DO) (M PLAN 20×, APO, LWD, NA = 0.42 Mitutoyo), an infinity-corrected tube lens (TTL200, Thorlabs), a long-pass filter (FEL0500, Thorlabs), and a 12-bit CCD camera (GS3-U3-28S4M, PointGrey). Additionally, for the scanning light-sheets, the exposure time of the CCD was adjusted in a way that multiple, but the same, number of scanning cycles of the scanning light-sheets could be recorded by the camera. The translation stage and the camera were synchronized and automated to capture images from arbitrary depths inside the sample.

**Figure 5:**
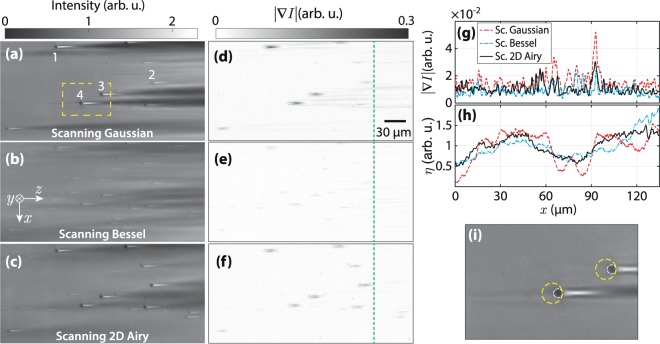
Experimentally captured LSFM images of non-fluorescent 4.4 *μ*m silica micro-beads embedded in a fluorescent gel with the scanning Gaussian (**a**), scanning Bessel (**b**), and scanning 2D Airy (**c**) light-sheets. (**d**–**f**) Modulus of the intensity gradient associated to (**a**–**c**), respectively. (**h**) Line profiles along *x* at a *z* position indicated by the dashed line in (**d**–**f**). (**g**) Plot of the final to initial intensity ratio($\eta$) along *x*-axis. (**i**) A magnified view of the dashed rectangle in (**a**); the areas around the selected beads, used for the calculation of local contrast, are shown by yellow dashed circles.

Captured images from a depth of 100 *μ*m inside the specimen, underneath the surface facing the DO, with different illuminating scanning light-sheets are depicted in Fig. 5(a–c). Each image is separately normalized to uniform fluorescent background intensity. Even without a quantitative assessment of the images it is obvious that the stripes are suppressed in the images with scanning non-diffracting light-sheets Fig. 5(b,c), while the image with the scanning Gaussian beam is seriously deteriorated by the presence of the stripes, although in this case the particles appear with a higher contrast in comparison to the images with scanning non-diffracting beams. For a quantitative assessment of the contrast in the images we calculated the local contrast for some of the beads. The selected particles are labelled with numbers 1–4, in Fig. 5(a). An ~80 *μ*m^2^ circular region around each particle, without including the corresponding stripes (as depicted by the dashed circles in Fig. 5(j)), was selected to calculate the local contrast defined by Eq. 3. The values of the local contrast for different illumination light-sheets and different particle numbers are given in Table 3. Clearly, the scanning Gaussian light-sheet provides the highest contrast for most of the particles, while the lowest contrasts for all particles are achieved with the scanning Bessel light-sheet, in a prefect agreement with the simulations. Besides, the contrast values for the image with the scanning 2D Airy light-sheet is comparable with that of a Gaussian light-sheet, while the effect of stripes is significantly reduced in the image with 2D Airy light-sheet. The modulus of the intensity gradients of the images in Fig. 5(a–c) are shown in Fig. 5(d–f). Higher values of the intensity gradient in the images imply stronger deterioration of the images by the stripes. Line profiles of the intensity gradient along $x$ direction at a single $z$ position, indicated by the dashed lines in Fig. 5(d–f), are plotted in Fig. 5(g) for all light-sheets, and the SDs of the intensity gradient over the indicated line are given in Table 4. It is seen that the stripe artifacts are significantly reduced in the image with scanning 2D Airy light-sheet.

**Table 3:**
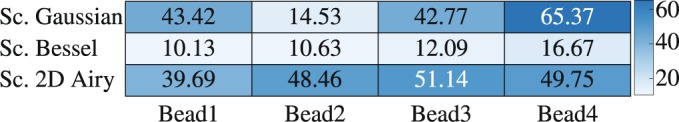
Local contrast of the micro-beads with different illumination light-sheets in the captured LSFM images.

**Table 4:** Standard deviation of the modulus of the intensity gradient over the line profile.

Illumination light-sheet	SD × 100
Scanning Gaussian	0.603
Scanning Bessel	0.336
Scanning 2D Airy	0.447

Furthermore, the ratio of the fluorescence intensity at the final propagation distance to that of the initial propagation distance, as $\eta \,(x)=\frac{I(x,z=290\,\mu {\rm{m}})}{I(x,z=0)}$, is selected as measure of the penetration depth of the light-sheets in the specimen. It should be noted that the waist of all three light-sheets were adjusted to be located at the middle of the FOV of the detection microscope at *z* = 145 *μ*m. In an ideal case, when the light-sheet is uniform within the sample, the ratio should have minor fluctuations around unit. For a comparison, the ratio $\eta (x)$ is plotted for images with different light-sheets in Fig. 5(h). Additionally, SDs of the final to initial intensity ratio in the images with different illumination light-sheets are given in Table 5. These data imply that the light-sheets with scanning non-diffracting beams, specially the scanning 2D Airy light-sheet, are more robust during the propagation in an inhomogeneous medium. The capability of resisting the effects of the inhomogeneities and absorbing structures in dense specimen, along with providing higher contrast of the structures in LSFM images, indicate that the scanning 2D Airy light-sheet is the optimal illumination option in LSFM.

**Table 5:** Standard deviation of final to initial intensity ratio (*η*) in the images with different illumination light-sheets.

Illumination light-sheet	SD
Scanning Gaussian	0.37
Scanning Bessel	0.33
Scanning 2D Airy	0.25

Further, we used the scanning 2D Airy light-sheet in 3D LSFM imaging of mammospheres of human breast cancer cell line, as real, dense, and inhomogeneous biological samples. For the sample preparation, the human breast cancer cell line MCF7 was cultured at 37 °C in RPMI 1640 (Sigma-Aldrich) containing 10% fetal bovine serum (Gibco), and 1% penicillin/streptomycin (Gibco). After the cells were 80% confluent, they were utilized for subsequent mammosphere formation. To create the mammospheres, the cells were detached and singled by adding 0.025% trypsin for five minute and then neutralized using complete media. 2 × 10^3^ cells were seeded in each well of a 96-well plate coated with agarose. 100 *μ*L of complete media was added to each well and it was changed every 2 days. Mammospheres were generated and allowed to form up to an average diameter of 200 *μ*m at day 5, and then they were harvested. The selected mammospheres were stained using Acridine orange (AO), a nucleic acid-selective fluorescent dye with an excitation maximum at 502 nm and an emission maximum at 525 nm (green) to monitor the nuclei of mammosphere-forming cells. Although the fluorescence excitation was not performed at 502 nm, but an efficient excitation was achieved with an excitation at 473 nm. The mammosphere were then immersed in a quartz cuvette (with dimensions of 1 × 1 × 4 cm^3^, and wall thickness of 1 mm) filled with a 1 mL of agarose gel, in such a manner that the mammosphere was held 150 *μ*m away from a corner of the cuvette facing the IO and the DO. The cuvette was placed in a water chamber with quartz windows, after the solidification of the agarose gel. A side of the chamber was placed adjacent to the IO (without a gap), and the other side was placed at a distance of 17.6 mm from the DO.

**Figure 6:**
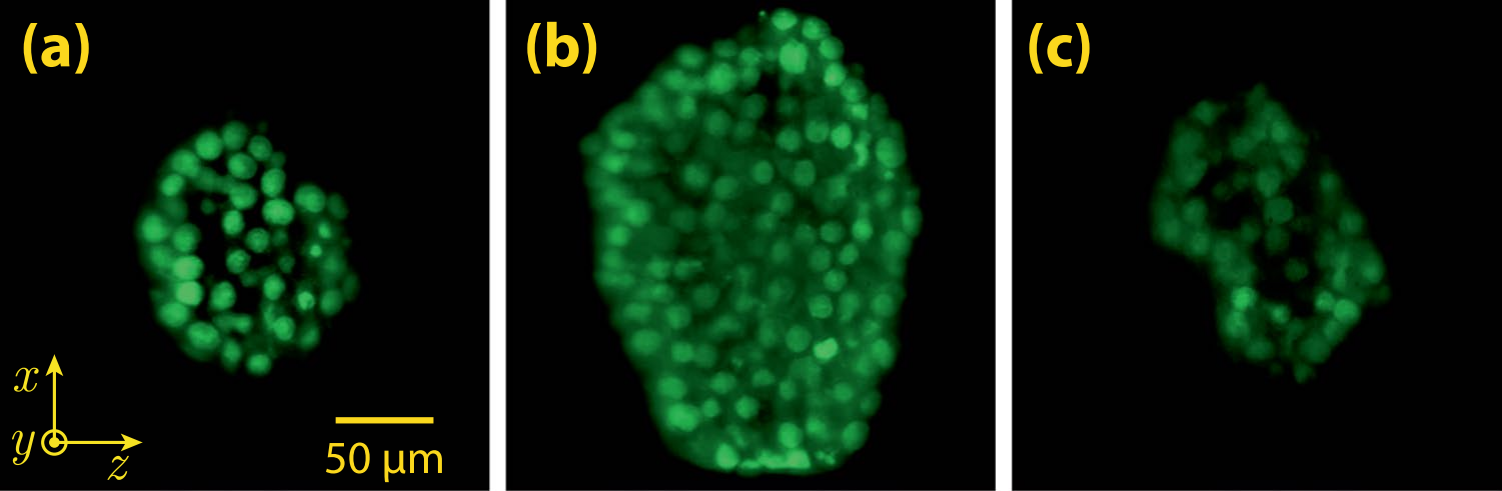
Experimentally captured cross-sectional LSFM images of a mammosphere, from three depths of 20, 120 and 220 *μ*m (**a**–**c**), respectively. The mammosphere was illuminated by a scanning 2D Airy light-sheet at a wavelength of 473 nm.

A 3D image stack of the mammosphere was captured with an exciting laser at a wavelength of 473 nm in the form of scanning 2D Airy light-sheet with a thickness (FWHM) of 2.8 *μ*m. The detection microscope, and also the illuminating light-sheet were fixed at their position, while the sample-containing cuvette was mounted on the motorized stage and displaced along −*y*-direction, to illuminate and image various depths of the sample. The motorized stage and the camera were synched in a way that after each step of displacement (by 200 nm), an image could be automatically captured. A long-pass filter with a cutoff wavelength of 500 nm was used to block the scattered laser beam. Finally, a home-developed deconvolution program based on Richardson-Lucy algorithm^25–27^ was used for deconvolution of the experimentally captured images with the illumination and detection PSFs. The illumination PSF was created by directly imaging the illumination beam profile along its propagation direction at several propagation distances with a step size of 800 nm, while the detection PSF was numerically calculated according to the Gibson-Lanny model^28^. The point spread function of the whole system (${{\rm{PSF}}}_{{\rm{sys}}}$) was generated by multiplication of the detection and illumination PSFs and neglecting the minor deviation of 2D Airy beam from a straight line, as a result of its parabolic trajectory. The deconvolution process was performed on the 3D stack of images, first on $x$-$z$ planes, and next on $y$-$z$ planes. The principle of the deconvolution algorithm along with relations for the detection PSF are given in the Methods section. Deconvolved cross-sectional ($x$-$z$) images from three depths in the mammosphere are shown in Fig. 6(a–c). These images are presented with a green colormap to resemble the color of fluorescence emission with a peak at 525 nm, although the captured images are monochromatic. Furthermore, a video of the maximum projection of the deconvolved 3D image stack of the sample, in which the mammosphere is rotated about $x$-axis is presented as a Supplementary Video. It is easily seen that the 3D image is indeed of very high quality, without artifacts, and not affected by the side lobes of the illuminating 2D Airy light-sheet.

**Figure 7:**
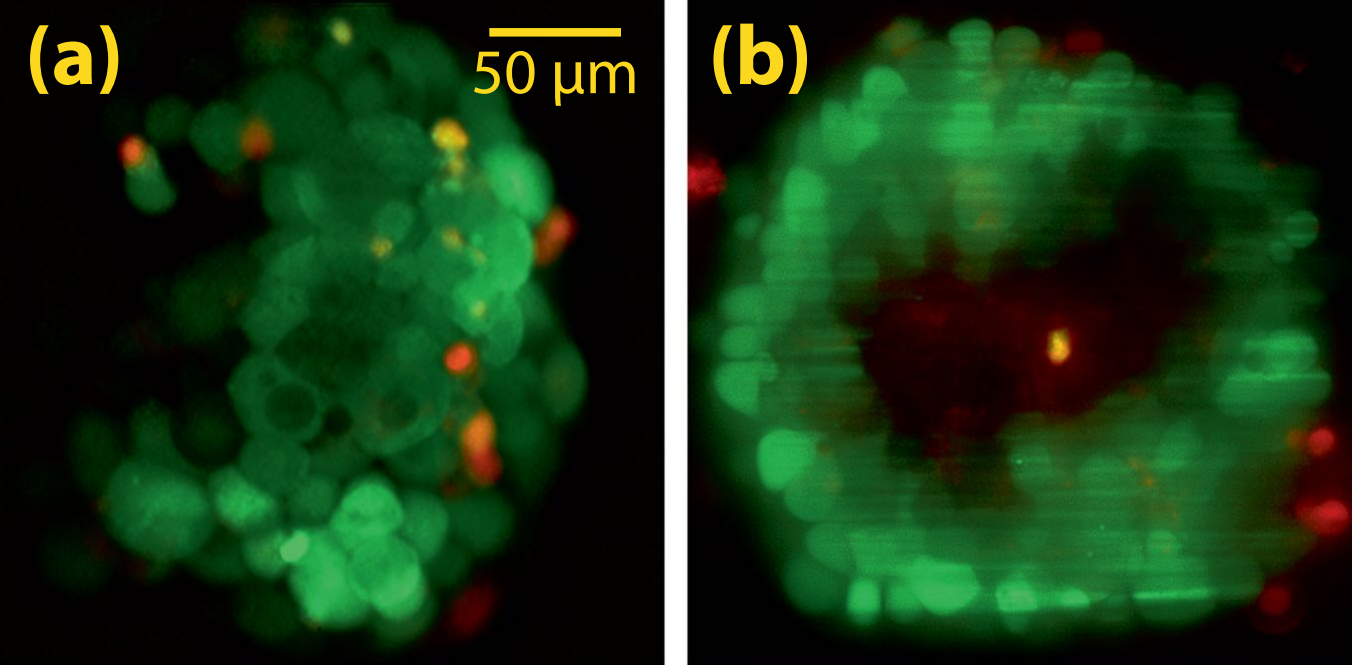
Experimentally captured LSFM images from a depth of 100 *μ*m inside mammospheres stained with two different dyes, and illuminated by scanning 2D Airy (**a**), and scanning Gaussian (**b**) light-sheets. In both cases, the excitations are separately performed with wavelengths 473 nm and 532 nm. For each illumination wavelength an image was captured, and the images were eventually merged to create a color-coded image. Here, the red color represents the emission solely from dead cells (excitation wavelength of 532 nm), while the green color originates from live cells (excitation wavelength of 473 nm).

Furthermore, two other mammospheres were prepared as described earlier and stained by calcein–acetoxymethyl (Calcein-AM; Sigma–Aldrich) and propidium iodide (PI; BioLegend). Calcein-AM is a cell permeable and non-fluorescent dye which is converted to green-fluorescent calcein, after hydrolysis by cellular esterases of live cells; hence, it is applicable to detect live cells with an excitation peak at 488 nm and maximum emission at 520 nm. On the other hand, PI is a fluorescent dye with an excitation peak at 535 nm and maximal emission at 617 nm (red) which is commonly used to detect dead cells. Therefore, dead and live cells could be discriminated by simultaneous or sequential excitation with proper wavelengths. For this purpose, scanning 2D Airy light-sheets with a similar thickness but different wavelengths (${\lambda }_{1}=473\,{\rm{nm}}$, and ${\lambda }_{2}=532\,{\rm{nm}}$) were separately used for LSFM imaging of the mammosphere. Two different long-pass filters with cut-off wavelengths of 500 nm, and 550 nm were used in the detection microscope for excitation laser beams at wavelengths of 473 nm, and 532 nm, respectively. A cross-sectional image from a depth of 100 *μ*m inside the mammosphere is shown in Fig. 7(a), where green and red colors represent live and dead cells, respectively. Interestingly, some cells simultaneously produce both green and red emissions which can possibly imply that they are dying. It should be noted that both images with double excitation were taken by the monochrome CCD; however, to make a distinction between the two emissions, the images are color-coded and merged. Additionally, for a comparison, a similarly stained mammosphere was imaged with the double-wavelength excitation scheme using scanning Gaussian light-sheets with a comparable thickness. A cross-sectional image of the mammosphere with such an illumination is demonstrated in Fig. 7(b), where image quality degradation is obviously noticeable. The stripe artifacts are easily observable in the image with scanning Gaussian light-sheet.

In conclusion, the effect of illumination in LSFM image quality with several light-sheets is investigated both experimentally and numerically. It is shown that static light-sheets in the form of 1D Gaussian and 1D Airy beams although provide higher contrasts in the images, are severely deteriorated by the inhomogeneities in the samples. On the other hand, it is shown that scanning light-sheets specially in the form of non-diffracting Bessel and Airy beams provide images with remarkably reduced artifacts. However, the scanning Bessel light-sheet and its 1D static counterpart (i.e. the cosine light-sheet) are unable to provide sufficient contrast for the structures in the samples. Scanning 2D Airy light-sheet, on the other hand, provides images with very high contrast (comparable to static light-sheets) along with markedly reduced stripe artifacts over a large FOV. This is attributed to the decreased spatial coherence, self-healing feature, asymmetric intensity distribution, and longer penetration depth of scanning 2D Airy light-sheets. The illumination scheme is utilized for both single- and double-wavelength excitation/detection of large, dense and inhomogeneous mammospheres of human breast cancer tumors and its shown that by a proper deconvolution of the 3D image stacks, high quality, artifact-free 3D images of such complex systems can be achieved by LSFM.

## Methods

### Numerical Simulations

For the simulations of LSFM imaging, presented in Figs. 2 and 3, BPM was implemented and used. The 3D sample was considered to be composed of non-fluorescent micro-beads with a refractive index of ${n}_{b}=1.6$ embedded in a uniform fluorescing medium with a refractive index of ${n}_{o}=1.4$. If the electric field envelope of the illuminating beam at a given propagation distance of $z$ is denoted by $U(x,y,z)$, the field envelope upon a propagation through the medium by a step size of Δ*z* is calculated as

4 $$\begin{array}{rcl}U(x,y;z+\Delta z) & = & {e}^{-i\Delta nk\Delta z}\\  &  & \times \,{ {\mathcal F} }^{-1}\{{e}^{-i\Delta z\sqrt{{k}^{2}-{k}_{\perp }^{2}}} {\mathcal F} \{U(x,y;z)\}\}\end{array}$$

where Δ$n={n}_{0}-{n}_{b}$ is the difference of the refractive index between the micro-beads and the surrounding medium, $k$ is the wavenumber in the surrounding medium, ${k}_{\perp }=\sqrt{{k}_{x}^{2}+{k}_{y}^{2}}$ denotes the magnitude of the transverse components of the wavevector, and ${\mathcal F}$, ${ {\mathcal F} }^{-1}$ represent 2D Fourier and inverse Fourier transformations, respectively. The simulations are performed with $\lambda =473\,{\rm{nm}}$, and a uniform grid spacing of $\Delta x=\Delta y=\Delta z=0.2\lambda$. The scanning light-sheets are achieved by displacing the illuminating beams along $x$-direction by a step size of $\delta x=360\,{\rm{nm}}$. Moreover, the simulations presented in Supplementary Fig. 5, are performed with the same parameters but with different size, and refractive indices of the micro-beads. The refractive index, size and 3D distribution of the micro-beads in both cases are presented in Supplementary Fig. 3.

### Born-Wolf model for the detection PSF in numerical simulations of the LSFM

In the numerical investigations, the effect of the detection microscope was taken into account by performing a convolution between the simulated fluorescence emission and point spread function of the detection that was calculated based on the Born-Wolf model. According to this model, in an aberration-free system, the light intensity distribution around the focus of the detection objective, that is defined as the point spread function of detection, ${{\rm{PSF}}}_{{\rm{\det }}}$, is given by^24^

5 $${{\rm{P}}{\rm{S}}{\rm{F}}}_{det}(r,y)=A{|{\int }_{0}^{1}{J}_{0}(k{\rm{N}}{\rm{A}}r\rho ){e}^{-i\frac{k}{2}{{\rm{N}}{\rm{A}}}^{2}{\rho }^{2}y}\rho d\rho |}^{2},$$

where $A$ is a constant, $k$ is the wavenumber of the emitted light, ${J}_{0}(\cdot )$ is the zeroth-order Bessel function of the first kind, $r=\sqrt{{x}^{2}+{z}^{2}}$ is the lateral radius on the plane of the detector ($x$-$z$ plane), $y$ is the axial distance between the focal plane and the plane of the detector, ${\rm{NA}}$ is the numerical aperture of the system, $\rho =r/a$ is the radial distance normalized to the aperture radius of the objective, $a$. ${{\rm{PSF}}}_{{\rm{\det }}}$ was calculated through direct integration with ${\rm{NA}}=0.42$, $\lambda =515\,{\rm{nm}}$. The transverse and longitudinal profiles of the calculated point spread function are shown in Supplementary Fig. 4.

### Gibson-Lannni model for the experimental detection PSF

Unlike the Born-Wolf model, in the Gibson-Lanni model, external aberrations such as the ones originating from the index mismatch between sample, coverslip, and immersion can be taken into account. Such aberrations are characterized by optical path length difference between a ray in an ideal system and a ray under the experimental condition. According to this model, the detection point spread function is given by^28^

6 $${{\rm{P}}{\rm{S}}{\rm{F}}}_{det}(r,y)=A{|{\int }_{0}^{1}{J}_{0}(kr{\rm{N}}{\rm{A}}\rho ){e}^{ik\Lambda }\rho d\rho |}^{2},$$

with

$$\begin{array}{rcl}\Lambda  & = & \left({y}_{s}-y+{n}_{i}\left(\,-\,\frac{{y}_{s}}{{n}_{s}}+\frac{{t}_{i}}{{n}_{i}}\right)\right)\sqrt{{n}_{i}^{2}-{{\rm{NA}}}^{2}{\rho }^{2}}\\  &  & +\,{y}_{s}\sqrt{{n}_{s}^{2}-{{\rm{NA}}}^{2}{\rho }^{2}}-{t}_{i}\sqrt{{n}_{i}^{2}-{{\rm{NA}}}^{2}{\rho }^{2}},\end{array}$$

where ${y}_{s}$ is the axial position of a fluorophore point source from the immersion layer, $y$ is the axial distance measured from the immersion layer, ${n}_{s}$ is the refractive index of the sample, ${t}_{i}$ and ${n}_{i}$ are the thickness and refractive index of the immersion layer, respectively. For simplicity, effects of the thin glass windows are neglected, and it is also assumed that the refractive index of the sample and that of the water are identical. The integral was directly calculated according to experimental configuration for imaging the mammospheres, with ${n}_{s}=1.33$, ${y}_{s}=6.8\,{\rm{mm}}$, ${t}_{i}=17.6\,{\rm{mm}}$, ${n}_{i}=1$, ${\rm{NA}}=0.42$, and $\lambda =525\,{\rm{nm}}$. Cross-sectional distributions of the calculated ${{\rm{PSF}}}_{{\rm{\det }}}$ are presented in Supplementary Fig. 7.

### Richardson-Lucy Deconvolution

With a given point spread function of the system, captured LSFM images could be properly deconvolved using the iterative Richardson-Lucy (RL) deconvolution algorithm. Among other deconvolution approaches, the RL algorithm is shown to be capable of sufficiently retrieving the distribution even in the presence of electronic noise of the detector. The RL iterative algorithm is expressed as

7 $${f}^{(m+1)}={f}^{(m)}\left({{\rm{PSF}}}_{{\rm{sys}}}^{{\rm{T}}}\,\ast \,\frac{{I}_{{\rm{image}}}}{{{\rm{PSF}}}_{{\rm{sys}}}\,\ast \,{f}^{(m)}}\right),$$

where $f$ is the fluorophore distribution, $m$ is the iteration number, ${I}_{{\rm{image}}}$ is the captured image, ${{\rm{PSF}}}_{{\rm{sys}}}$ is the point spread function of the whole system, superscript ${\rm{T}}$ implies matrix transposition, and the symbol $\ast$ indicates a 2D convolution process.

## Supplementary information

Supplementary Information.

Supplementary Information2.
