# Supplementary material for: Light-Sheet Fluorescence Microscopy with Scanning Non-diffracting Beams
Source: Sci Rep. 2020 May 22;10:8501. doi: 10.1038/s41598-020-63847-2 (PMC7244762; doi:10.1038/s41598-020-63847-2)
Supplement: Supplementary file 1 — Supplementary Information. [file 41598_2020_63847_MOESM1_ESM.pdf]

Supplementary Materials

Light-Sheet Fluorescence Microscopy with Scanning Non-diffracting Beams

Hosein Kafian[1], Meelad Lalenejad[1], Sahar Moradi-Mehr[2], Shiva Akbari Birgani[2], and Daryoush Abdollahpour[1, 3, *]

[1]Department of Physics, Institute for Advanced Studies in Basic Sciences (IASBS), Zanjan 45137-66731, Iran
[2]Department of Biology, Institute for Advanced Studies in Basic Sciences (IASBS), Zanjan 45137-66731, Iran
[3]Optics Research Center, Institute for Advanced Studies in Basic Sciences (IASBS), Zanjan 45137-66731, Iran

*Corresponding author: dabdollahpour@iasbs.ac.ir

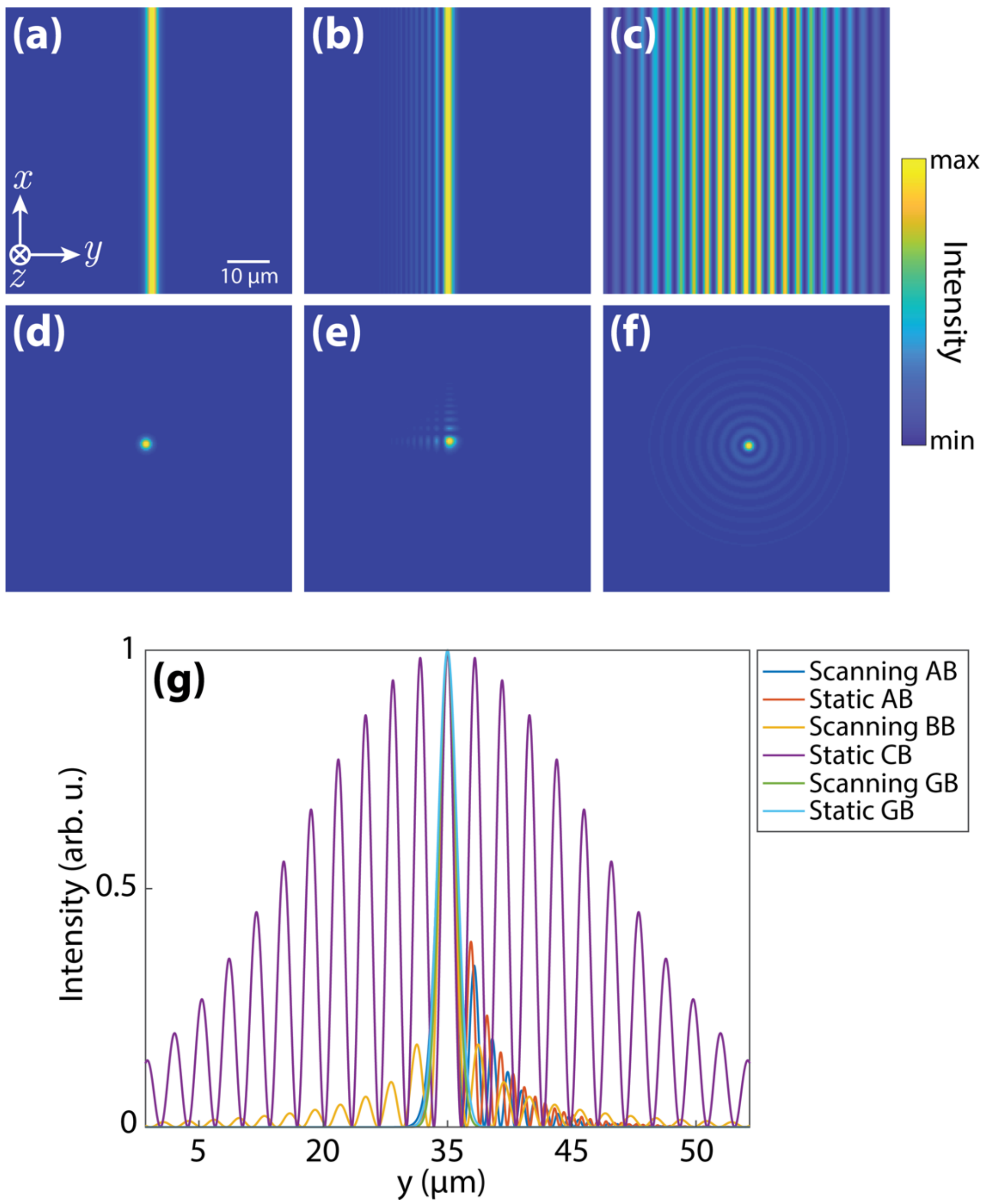

Supplementary Figure 1 | Simulated Beam Profiles. Transverse (x-y) normalized intensity profiles of the simulated beams at their waist for 1D Gaussian (**a**), 1D Airy (**b**), cosine (**c**), 2D Gaussian (**d**), 2D Airy (**e**), and Bessel (**f**) beams. The static light-sheets were formed in the x-z plane by 1D Gaussian, cosine, and 1D Airy beams while the scanning light-sheets were formed in the same plane by gradually displacing 2D Gaussian, 2D Airy, and Bessel beams along x-axis. Normalized profiles of the light-sheets along y-axis are shown in (**g**). Here, AB, GB, CB, and BB indicate Airy beam, Gaussian beam, cosine beam, and Bessel beam, respectively.

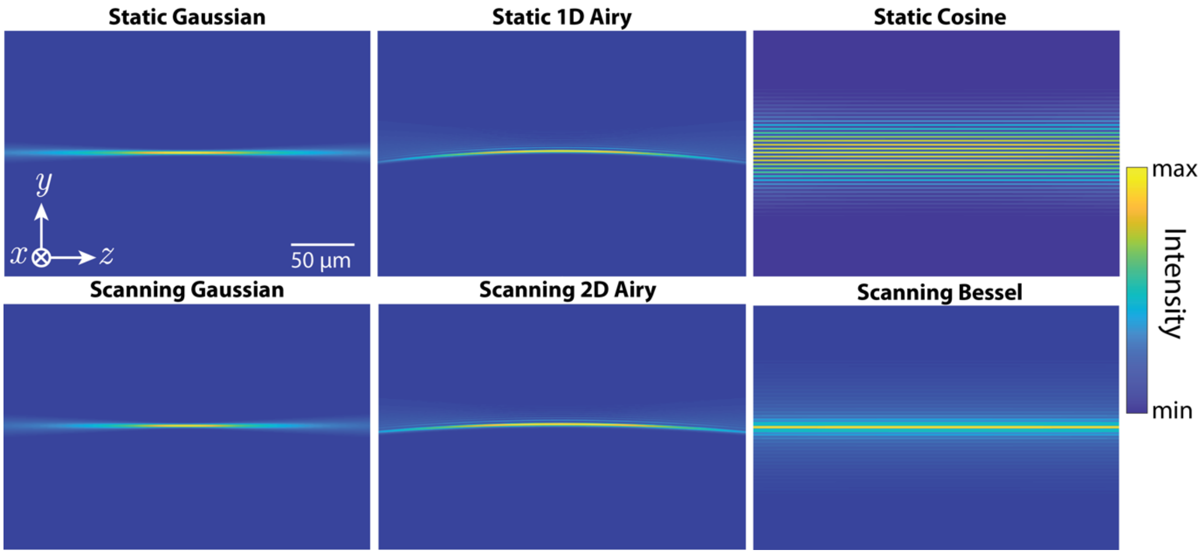

Supplementary Figure 2 | Simulated Light-Sheet Profiles along Propagation. Intensity profiles of the simulated light-sheets along their propagation direction. The thickness of the Gaussian light-sheet is increased by a factor four, with respect to the waist, at the edges of the FOV along z-axis, while the cosine and Bessel light-sheets are propagation-invariant in the range, and the thickness of the Airy light-sheets are slightly increased by 13%.

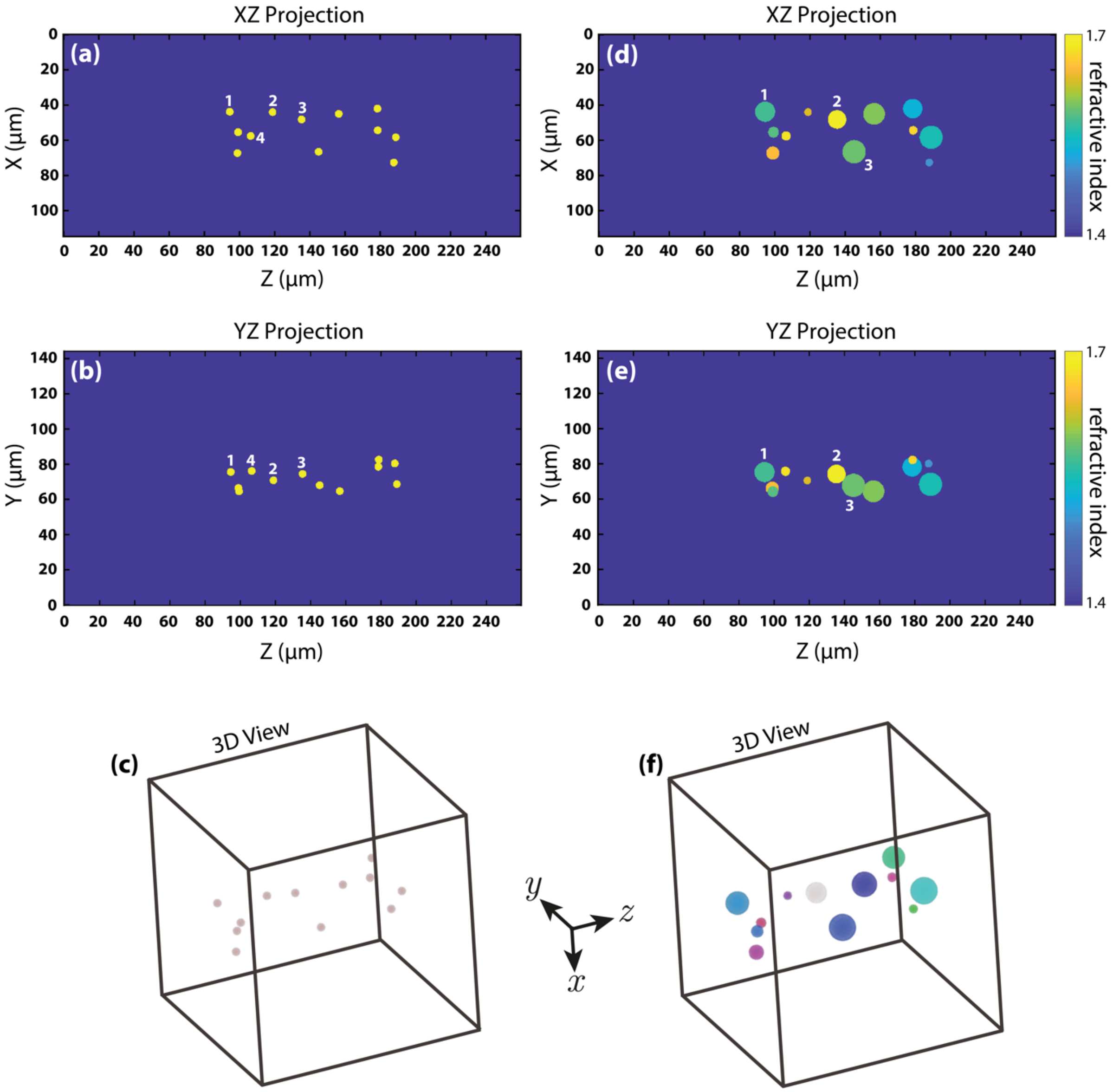

Supplementary Figure 3 | 3D Particle Distribution Used in Numerical Simulations of LSFM. The particle distribution whose various views are depicted in (**a-c**) are used for numerical simulations presented in Fig. 2 and Fig. 3. In these cases, the particles have the same diameter of 4.4 µm and refractive index of 1.6. The particle distribution whose various views are depicted in (**d-f**) are used for numerical simulations presented in Supplementary Figure 5; here the particle sizes (diameter) and refractive indices were randomly selected in the ranges of 4.4 µm - 13.2 µm, and 1.5-1.7, respectively. The numeric labels in (**a**, **b**) indicate the randomly selected particles for local contrast calculations reported in Table II. Similarly, the numeric labels in (**d**, **e**) indicate the randomly selected particles for local contrast calculations reported in Supplementary Figure 5(**i**).

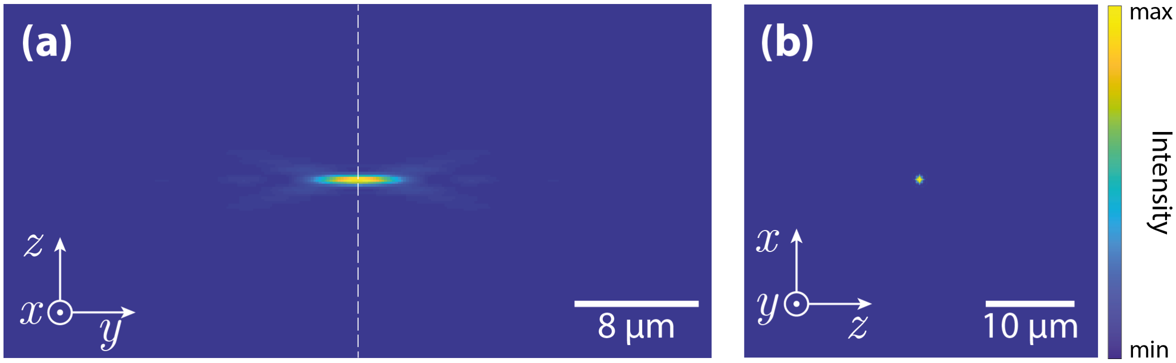

Supplementary Figure 4 | Calculated Detection PSF Based on Born-Wolf Model. (y-z) (**a**), and (x-z) (**b**) cross-sections of the calculated detection PSF based on Born-Wolf model. The (x-z) cross-section corresponds to the focus of the PSF indicated by the dashed line in (**a**). The calculated PSF with λ=515 nm, and NA=0.42, was used to simulate the effect of the detection microscope in numerical simulations of LSFM imaging of the micro-beads.

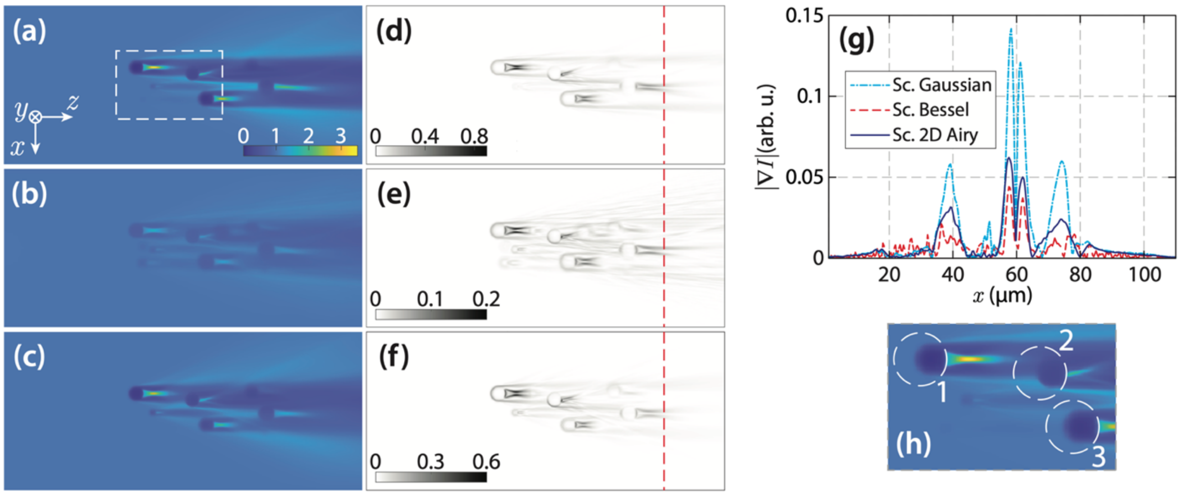

(i)

	Bead1	Bead2	Bead3
Sc. Gaussian	89.39	69.51	84.98
Sc. Bessel	18.32	17.50	19.82
Sc. 2D Airy	81.71	61.81	51.23

Supplementary Figure 5 | Numerically Simulated LSFM Images of Particles with Random Sizes and Refractive Indices. Numerically simulated LSFM images of non-fluorescent micro-beads, with different sizes and refractive indices, embedded in a uniform fluorescent medium, illuminated with the scanning Gaussian (**a**), scanning Bessel (**b**), and scanning 2D Airy (**c**) light-sheets with identical thicknesses. The 3D distribution of the particles, their sizes and refractive indices are shown in Supplementary Figure 3 **(d-f)**. (**d-f**) Modulus of intensity gradient corresponding to (**a-c**), respectively. (**g**) Line profiles of the intensity gradients over the indicated dashed lines in (**d-f**) along x-axis. (**h**) A magnified view of the dashed rectangle in (**a**); three selected particles for local contrast calculations are labeled with numbers 1-3. (**i**) Calculated local contrast for the three particles indicated in (**h**). For calculations of local contrast, a 450 μm^2 circular regions around each particle (without including the stripe artifact after individual particles) were selected.

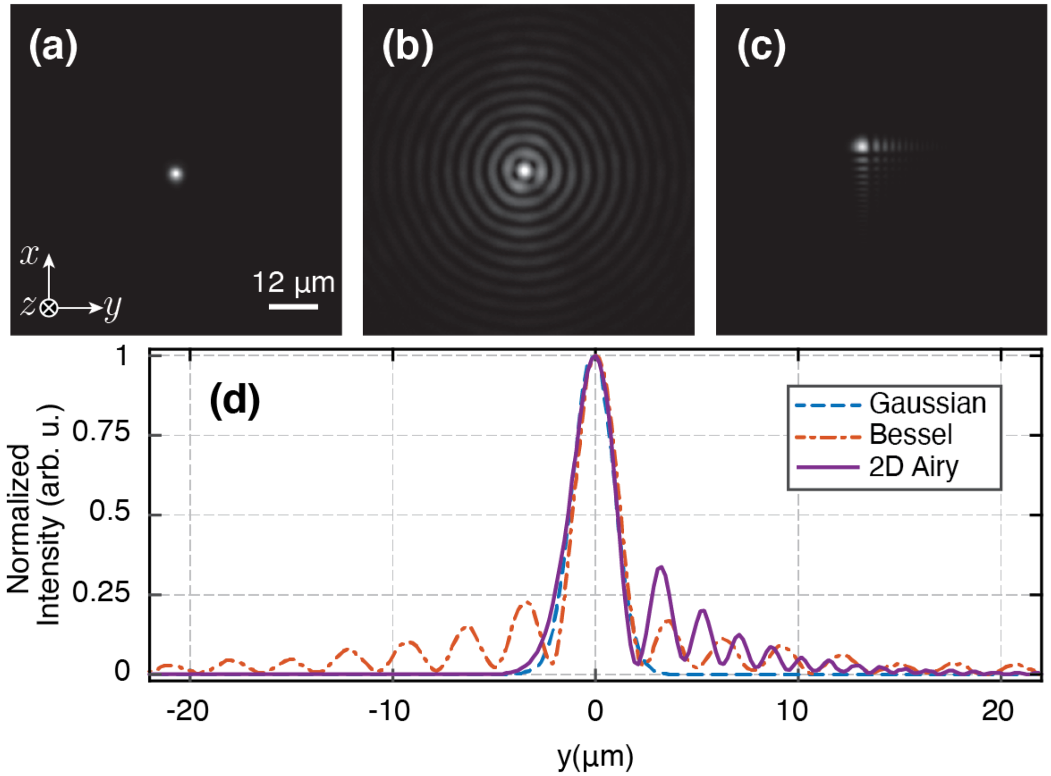

Supplementary Figure 6 | Transverse Profiles of the Experimentally Generated Light-Sheets. Transverse (x-y) normalized intensity profiles of the experimentally generated beams at their waist (i.e. at the focal plane of the IO). (**a**) 2D Gaussian, (**b**) Bessel, and (**c**) 2D Airy beams. (**d**) Normalized line profiles of the beams along y-axis.

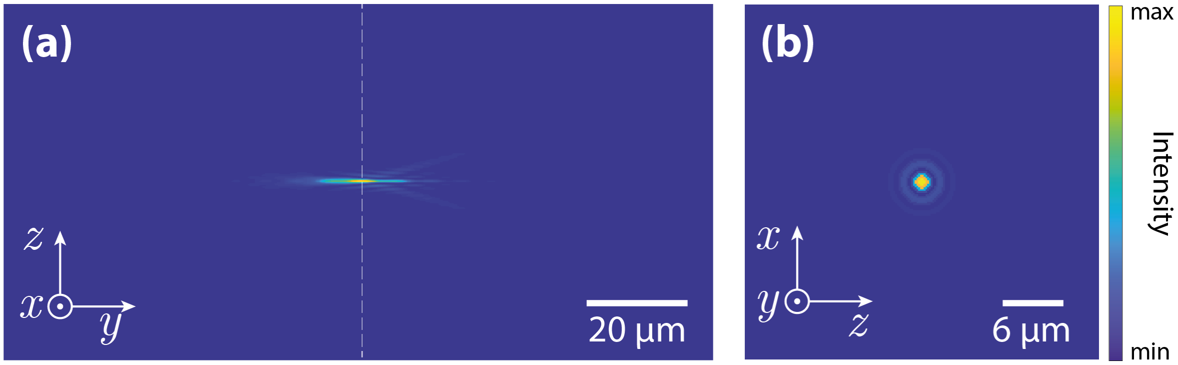

Supplementary Figure 7 | Calculated Detection PSF based on Gibson-Lanni Model. (y-z) (**a**), and (x-z) (**b**) cross-sections of the calculated detection PSF based on Gibson-Lanni model. The (x-z) cross-section corresponds to the focus of the PSF indicated by the dashed line in (**a**). The calculated PSF with λ=525 nm, and NA=0.42, was used to create the PSF of the whole system for deconvolution of the experimentally recorded 3D image stacks of the mammospheres.
